# Supplementary material for: Phytochromes facilitate social behaviour in marine diatoms
Source: Nat Commun. 2026 Mar 10;17:3766. doi: 10.1038/s41467-026-70219-3 (PMC13106708; doi:10.1038/s41467-026-70219-3)
Supplement: Supplementary file 1 — Supplementary Information [file 41467_2026_70219_MOESM1_ESM.pdf]

Supplementary Information for

Phytochromes facilitate social behaviour in marine diatoms

Joan S. Font-Muñoz[1,2*], Marianne Jaubert[3], Marc Sourisseau[2], Idan Tuval[1], Benjamin Bailleul[3], Carole Duchêne[3,4], Gotzon Basterretxea[1], Angela Falciatore[3]

*Joan Salvador Font Muñoz
Email: jfont@imedea.uib-csic.es

This PDF file includes:

Supplementary Figures S1 to S4

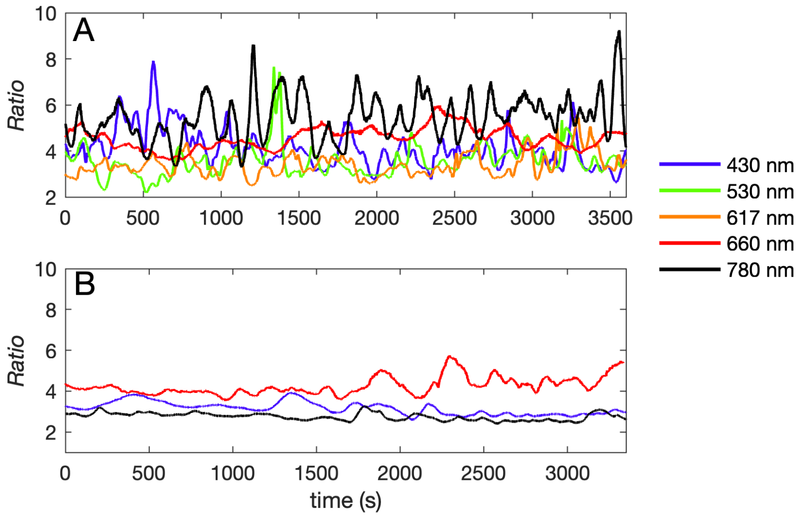

Figure S1. **Collective response to light in *P. tricornutum*.** Time series of *Ratio* obtained in *P. tricornutum* experiments performed with A) wild-type strains and B) *dph*-knockout lines. Colors indicate the wavelength with which cells were illuminated (430, 530, 617, 660, and 780 nm).

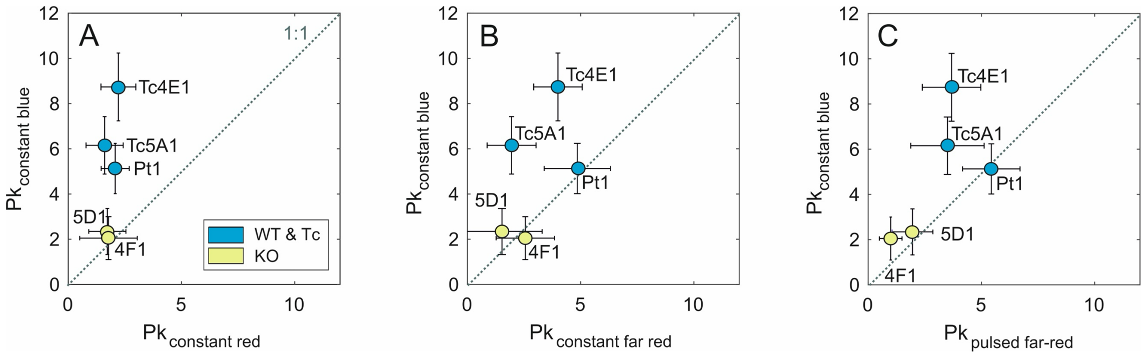

Figure S2. **Collective responses mediated by phytochromes.** A) Peak amplitude of the power spectrum for experiments with constant blue light compared to constant red light. B) Peak amplitude of the power spectrum for experiments with constant blue light compared to constant far-red light. C) Peak amplitude of the power spectrum for experiments with constant blue light compared to pulsed far-red light. Data are shown as mean ± SD (n = 3 independent biological replicates).

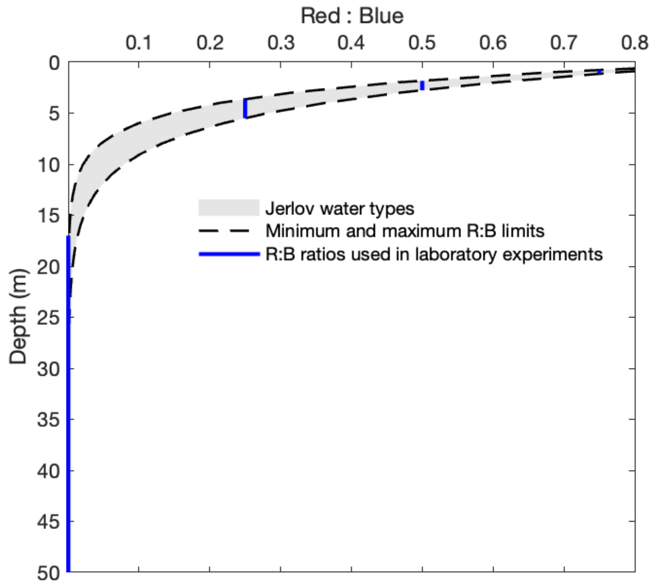

Figure S3. **Vertical distribution of the Red:Blue light ratio in the sea**. The grey-shaded region indicates the range of variation for different Jerlov water types (values from Table V of Austin & Petzold, 1986). The blue lines indicate the ratio used in the laboratory experiments shown in Figure 2.

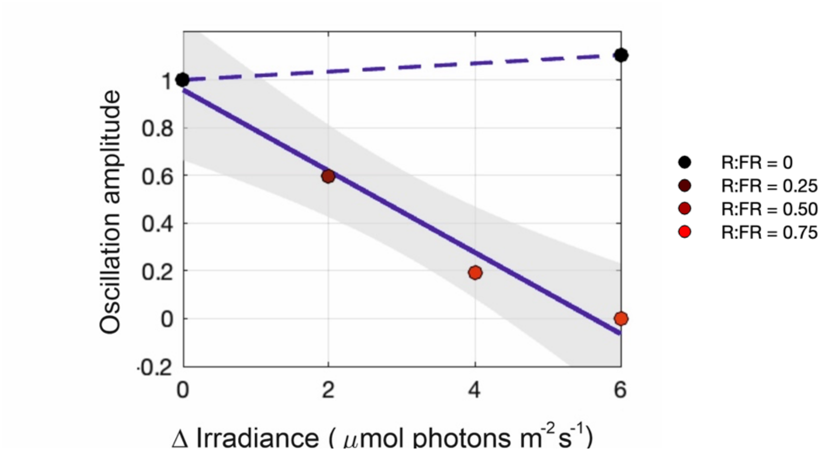

Figure S4. **Diatom response to continuous exposure to red and far-red light**. Normalized oscillation amplitude for experiments with enhanced light (under *FR l*ight background of 8 µmol photons m^{-2} s^{-1}) at different ratios of *R:FR* light and the controls with *FR* light. Blue lines show the linear fits to the experimental data; shaded bands indicate the corresponding 95% confidence intervals. The color band indicates the 95% confidence band. Dot colors indicate the ratios similarly to those in Figure 2B.
