# Supplementary material for: Phytochromes facilitate social behaviour in marine diatoms
Source: Nat Commun. 2026 Mar 10;17:3766. doi: 10.1038/s41467-026-70219-3 (PMC13106708; doi:10.1038/s41467-026-70219-3)
Supplement: Supplementary file 3 — Description of Additional Supplementary Files [file 41467_2026_70219_MOESM3_ESM.pdf]

Description of Additional Supplementary Files

Supplementary Data 1

Description: Raw and processed data, including power spectra of *P. tricornutum* (WT and DPH KO) under continuous light at multiple wavelengths (430, 530, 617, 660, 780 nm), DPH activation/deactivation spectra.

Supplementary Data 2

Description: Raw and processed data on diatom responses to continuous blue and red light, including mean oscillation periods, normalized oscillation amplitudes under different R:B light ratios, and time series of normalized ratios for experimental and control conditions.

Supplementary Data 3

Description: Time series and power spectra of natural fluorescence emission from WT and DPH knockout *P. tricornutum* under 430 nm light.

Supplementary Data 4

Description: Power spectra of Ratio measurements from pulsed and continuous light experiments for WT, Tc, and DPH knockout strains under red and far-red light.

Supplementary Data 5

Description: Time series of Ratio measurements in *P. tricornutum* for wild-type and DPH knockout strains under different wavelengths (430, 530, 617, 660, 780 nm).

Supplementary Data 6

Description: Peak amplitudes of power spectra for *P. tricornutum* under constant blue, red, and far-red light, and pulsed far-red light, showing phytochrome-mediated collective responses.

Supplementary Data 7

Description: Vertical distribution of the Red:Blue light ratio in the sea.

Supplementary Data 8

Description: Raw and processed data on diatom responses to continuous red and far-red light, including mean oscillation periods, normalized oscillation amplitudes under different R:FR light ratios, and time series of normalized ratios for experimental and control conditions.
